# A system of quantities from software metrology^✩^

**DOI:** 10.1016/j.measurement.2020.108435

**Published:** 2021

**Authors:** David Flater

**Affiliations:** National Institute of Standards and Technology, 100 Bureau Dr, Gaithersburg MD 20899, USA

**Keywords:** Software, International System of Quantities, International System of Units, Counted quantity, Dimensionless quantity, Bit

## Abstract

International Organization for Standardization (ISO)/International Electrotechnical Commission (IEC) 80000, the International System of Quantities, collects and organizes the most important physical quantities into a coherent system of quantities whose foundation for measurements is the International System of Units (SI). This short communication outlines a report that, in a similar fashion, collects and organizes the most important quantities used in measurements performed on software artifacts, focusing on software as a product rather than its development process.

## 1. Introduction

“Digitalization”, in the sense of improving the application of information technology in the work of metrology, has been widely embraced. The reverse – improving the application of metrology in the work of information technology – has received less attention.

Software is a large part of information technology. Although dozens of books and thousands of articles about software measurement have been published, the relationship of software measurement practices to conventional metrology has never been clear. The nearest thing to an “International System of Units (SI) for software” currently existing in the library of applicable standards is part 13 of the International Organization for Standardization (ISO)/International Electrotechnical Commission (IEC) International System of Quantities (ISQ). IEC 80000-13:2008, Quantities and units—Part 13: Information science and technology [1] includes a few quantities that apply to software, but its actual focus is communications technology.

A National Institute of Standards and Technology (NIST) interagency report, NISTIR 8289, targets this gap in applicable standards [2].^1^ The full text is freely available through the DOI. This short communication provides an outline of the report. Section 2 spells out the relevance and importance of the work. Section 3 clarifies the scope of measurements that it covers. Section 4 explains the important role of counted quantities. Section 5 addresses units and traceability. Section 6 describes how the concept of dimensions applies. Finally, Section 7 wraps up with observations on the standardization process.

## 2. Motivation

The SI system began in the 19th century and has continued to evolve ever since, growing in response to discoveries such as electricity and optics and changing to incorporate more precise measurements. But a new kind of product was invented in the middle of the 20th century to which the SI system has not yet responded: software.

Software has greatly transformed products, services, organizations, and human societies. Although commerce still is dominated by the exchange of physical goods, that exchange and nearly every other commercial process has been revolutionized by, and has become dependent on, software. As a result, although the economic benefits of information technology are reckoned in trillions of US dollars [3], mistakes that are attributable to the lack of reliable measurements of software can incur costs on a similar scale.

Since the established discipline of metrology has proven effective in so many applications, it may not be obvious why it was not applied to software measurement. The primary reason is that the digital space in which software exists has a different nature than physical space. Where physical measurement begins with ratios of continuous quantities, software measurement instead begins with discrete counts. Where physical metrology has chains of ratio comparisons, software metrology instead has definitions that are instantly realized everywhere they are needed. While there is counting in physical measurement, it is on the edge of the scope of physical metrology. In software measurement, this situation is reversed: most quantities are determined through counting, and the ones that are not are exceptional.

Many *ad hoc* software metrics have been defined and used. But when neither the methods of established metrology nor any comparable alternative are applied, the outcome is metrics and procedures that do not meet expectations for metrological rigor and results whose meaning and significance are unclear. Lack of consensus on the definitions of measurands has led to a proliferation of realized quantities, and lack of validated measurement methods has led to the production of quantitative indicators by any plausible means.

By adopting a more rigorous foundation and replacing *ad hoc* accretion with systematic structure, NISTIR 8289 sets a better direction for the future evolution of software metrology.

## 3. Scope

The word “software” covers a lot of territory, either directly or indirectly. A notional software attribute can be different things depending on which artifact or process serves as the object of measurement. The objects of measurement that are in scope of NISTIR 8289 include the following:

- Architecture and design;
- Requirements;
- Specification;
- Algorithm;
- Implementation (source code or script);
- Executable (binary or bytecode); and
- Execution (of binary, bytecode, or script).

The software *development process* is the object of measurement in a lot of related work that focuses on forecasting the time and effort required to complete a software development project—these metrics are not in the scope of NISTIR 8289. Neither does NISTIR 8289 address software being one component of an instrument intended to measure a physical property [4] or software used to model, simulate, or predict physical properties, sometimes called *virtual measurement* (e.g., computational quantum chemistry)—in both cases, the object of measurement is not the software itself but something else.

NISTIR 8289 organizes the relevant quantities into the following categories:

- Countable entities and events, as described in Section 4;
- Basic quantities, which include physical quantities, quantities related to resources, processing, and transmission, and metrics from graph theory;
- Compatibility metrics, which characterize the environments in which software can be used;
- Algorithm metrics, which characterize the behaviors or intrinsic properties of algorithms;
- General design and implementation metrics, which characterize engineering properties of all kinds of software;
- Object-oriented design and implementation metrics, which more precisely characterize engineering properties of object-oriented software specifically;
- Testability (test coverage) metrics, which characterize the extent to which software can be tested or has been tested; and finally,
- Security metrics, which characterize the degree to which software mitigates or fails to mitigate risks inherent in its use.

## 4. Countable entities and events

In software metrology, nearly every quantity that is not actually a physical quantity is determined through counting—either directly, as in the case of a counted quantity (e.g., an amount of data, such as 1 GB), or indirectly, as in the case of a quantity that is derived as a function of counted quantities (e.g., Martin’s relational cohesion, which is the ratio of a number of relationships to a number of classes [2, §8.2]). The system of quantities for software metrology therefore is built upon definitions of the entities and events that are most commonly counted. NISTIR 8289 devotes a chapter to this important foundation, quoting definitions from existing references as often as possible.

Non-physical software metrics that are not derived from simple counts do exist. For example, if there is any way to know what the ideal output of an algorithm would be, even if that output is a complex object such as a digital image, a mathematical function can be used to quantify the “error” or distance between the actual output and the ideal output. There are also some metrics where a value from an ordinal scale is assigned based on an assessment that includes qualitative factors.

## 5. Units and traceability

The SI brochure [5] classifies most software metrics as dimensionless quantities for which the measurement unit is one. Associated rules and style conventions specify that they should be quoted as plain numbers with a careful description of what they indicate. Software metrology thus far has mostly complied, accidentally, by neglecting to identify dimensionless units in most cases; but the practice has proven to be harmful. The absence of a visible unit symbol obscures the definition of the measurand. As a result, derived quantities are in use that mix different kinds of counts in suspicious ways, such as by adding call counts squared to a ratio of variables per call to produce a combined measure [2, §7.10].

To avoid contributing to this problem, NISTIR 8289 follows a model that extends the interpretation of dimensionless quantities by introducing “extended units” [6]. In the simplest case, this means that counts identify the type of entity or event counted [7]. In less simple cases, it means that derived quantities now have algebraically determined units that shed new light on the natures of those quantities (and perhaps on their validity).

The SI brochure states that counts are traceable to the SI via the special unit one and “appropriate, validated measurement procedures” [5, §2.3.3]. But in general, counting involves characterizing what is being counted (say, lines of code), and this characterization involves a standard (definition of line of code) that is not part of the SI. For an automated count of digital entities, the algorithm used defines the measurement standard for the entities to be counted; it is an operationalized definition. Uncertainty that arises in the measurement process is attributable mostly to the identification of the entities, not the enumeration that occurs once such identification has been made.

## 6. Dimensions

For most counted quantities, there is only one obvious unit to use: the entity or event counted. But the coexistence of multiple “natural units” of data (bits, bytes, and occasionally words) means that often it is less misleading to cite *dimension data* than to specify any such unit.

In physical metrology, NIST has proposed that angle be included as an SI dimension with the radian as its coherent unit of measure and the cycle as a non-coherent unit that is equal to 2*π* radians. In parallel fashion, we sometimes find it convenient to think of data as an added dimension with the bit as its coherent unit of measure and the byte as a non-coherent unit that is equal to 8 bits.

Stating that a quantity has the dimension of data, rather than the units of bits or bytes, makes it clear that the choice of unit is not an essential part of the definition of the quantity. One can use other counting units, such as data structures of a particular type that can be reduced to a count of bits, without losing traceability.

The dimensions that proved useful in describing software quantities were named as time, data, information, and work. Other “effective” dimensions corresponding to the many kinds of quantities described in NISTIR 8289 can easily be posited; however, in most cases, there is nothing to be gained by doing so. The units and/or scale of the result provide complete information.

## 7. Conclusion

While both structural differences and social phenomena have prevented software metrology from merging with physical metrology, physical metrology nevertheless encounters challenges with dimensionless quantities that software metrology has been forced to address.

In current practice, many of the elementary quantities of software measurement are multiply-defined and/or ill-defined. Improving and standardizing the definitions of these base quantities is within the traditional scope of international standards work. The transition from descriptive (following the practice) to prescriptive (normative) references requires consensus, and the proper venue for such a consensus to emerge is international standards.

## Footnotes

1 The report was indeed submitted to the standards process. History is provided in [2, §1.4].
